# On Prolapsus Uteri

**Published:** 1849-07

**Authors:** 


*On Prolapsus Uteri.* By Professor Hohl.—Professor Hohl believes that some very erroneous notions prevail as to the *causes* of this occurrence, and that some light may be thrown upon the subject by considering the changes of position which the uterus normally undergoes at different periods of life. In the mature fetus the uterus projects considerably beyond the pelvis; and it is only when it has acquired its completed shape and size at puberty, that it is found entirely within the cavity. At the commencement of the menstrual cycle it retains its position, or even rises still higher in the pelvis, while at the termination of this it again sinks, with the loss of blood, in stout young women. In women who seldom or never bear children it sinks still deeper, as it does, too, after the menstrual functions have ceased. In pregnancy the organ rises remarkably, and M. Hohl denies the correctness of the statement that it sinks lower in the pelvis after the second month, the apparent sinking being due to the turgescence of the organ, and especially of its cervix. After delivery the uterus remains high up in the abdomen, and only gradually resumes its ordinary position. In old women it is found deep in the pelvis.

The production of prolapsus is not dependent upon the condition of the vagina, and the ligaments of the uterus. The vital power of the organ may be said to maintain it in position. When this is augmented the uterus is raised, while, when it is diminished or lost, it descends.—Other organs, and indeed the whole body, in like manner exhibit strength and power proportionate to their *turgor vitalis.* The increase of the vital activity of the uterus during its development and growth, as also during menstruation and in pregnancy, is attended with elevation of the organ, which sinks again when these conditions prevail no longer. So far from allowing that the prolapse results from defective supporting power of the vagina, we may rather regard the uterus as supporting the vagina, and prolapsus of the latter may occur without any prolapsus uteri.

Thus the author refers the production of prolapsus to a preceding or coexisting condition of health, giving rise to a diminished vitality. This explains why we so seldom meet with the disease in young healthy women; while we know that whatever favors the relaxation of the genital system, and lowers the tone of the fibre, acts predisposingly,—the germ of the evil being found in the puerperal condition, when the uterus, after having been high up in the abdomen, sinks down into the lesser pelvis.

Although prolapsus may be secondarily produced by other affections, as tumors of the belly, prolapsus vaginæ, cystocele, &c. &c., yet far more frequently a change in the direction, rather than in the position of the organ then takes place; and even while the portion of the rectum in connexion with its posterior wall may prolapse entirely, the uterus may retain its normal position.

There may be a diseased condition of the economy in general, or of the uterus in particular, upon which depends this extinction or diminution of its vital power; and accordingly as this is or is not curable, will depend whether a cure of a prolapsus is apparant or real; as mere reposition with mechanical support is not a cure. In some diseases which are attended with an increased activity of the uterus, there is a rising of the organ in the pelvis, as puerperal fever, hydrometra, &c. Disease of the ovaries does not produce any sinking of the organ; nor do tumors or indurations of its substance as long as they are in process of development, nor until they have interrupted its functions, or weighed it down by their great bulk. Polypi also seldom give rise to prolapsus.

*Treatment.*—Common as is the disease, a radical cure is seldom accomplished. The indications are to remedy the defective or disordered condition of the general vital powers, or of those of the uterus in particular. The author especially warns us against the continued use of injections, and the too early employment of pessaries.—When the vital power of the sexual system or uterus is exhausted in consequence of age, over-stimulus, or incurable disease, mere palliative treatment should be employed.—*Zeitschrift für Geburtskunde.—Medico-Chirurg. Review.*

